# Supplementary material for: Resolving the full spectrum of human genome variation using Linked-Reads
Source: Genome Res. 2019 Apr;29(4):635–45. doi: 10.1101/gr.234443.118 (PMC6442396; doi:10.1101/gr.234443.118)
Supplement: Supplemental Material [file supp_gr.234443.118_Supplemental_longranger-2.2.zip › longranger-2.2.tar.bz2!/lr2.2-public/lib/stan/cmdstan/stan/doc/api/html/rev_2functions_2tan_8hpp_source.html]

Stan: src/stan/agrad/rev/functions/tan.hpp Source File


|  |  |
| --- | --- |
|  | Stan  2.5.0  probability, sampling & optimization |


- Main Page
- Related Pages
- Namespaces
- Classes
- Files

- File List
- File Members

All Classes Namespaces Files Functions Variables Typedefs Enumerations Enumerator Friends Macros Pages

- src
- stan
- agrad
- rev
- functions

tan.hpp

Go to the documentation of this file.

1 #ifndef STAN\_\_AGRAD\_\_REV\_\_FUNCTIONS\_\_TAN\_HPP

2 #define STAN\_\_AGRAD\_\_REV\_\_FUNCTIONS\_\_TAN\_HPP

3

4 #include <cmath>

5 #include <stan/agrad/rev/var.hpp>

6 #include <stan/agrad/rev/internal/v\_vari.hpp>

7

8 namespace stan {

9  namespace agrad {

10

11  namespace {

12  class tan\_vari : public op\_v\_vari {

13  public:

14  tan\_vari(vari\* avi) :

15  op\_v\_vari(std::tan(avi->val\_),avi) {

16  }

17  void chain() {

18  avi\_->adj\_ += adj\_ \* (1.0 + val\_ \* val\_);

19  }

20  };

21  }

22

50  inline var tan(const var& a) {

51  return var(new tan\_vari(a.vi\_));

52  }

53

54  }

55 }

56 #endif

stan::agrad::tan

fvar< T > tan(const fvar< T > &x)

**Definition:** tan.hpp:14

stan::agrad::var::vi\_

vari \* vi\_

Pointer to the implementation of this variable.

**Definition:** var.hpp:40

v\_vari.hpp

var.hpp

stan::agrad::var

Independent (input) and dependent (output) variables for gradients.

**Definition:** var.hpp:27


---

[ Stan Home Page ]

*© 2011–2014,
Stan Development Team.*
